# Prevalence of High-Risk Genotypes of Human Papillomavirus: Women Diagnosed with Premalignant and Malignant Pap Smear Tests in Southern Ecuador

**DOI:** 10.1155/2017/8572065

**Published:** 2017-06-22

**Authors:** Paola Dalgo Aguilar, Cisne Loján González, Ana Córdova Rodríguez, Katherine Acurio Páez, Ana Paulina Arévalo, Jana Bobokova

**Affiliations:** ^1^Departamento de Ciencias de la Salud, Sección Genética Humana, Microbiología y Bioquímica Clínica, Universidad Técnica Particular de Loja, Loja, Ecuador; ^2^Sociedad de Lucha Contra el Cáncer, Hospital SOLCA Núcleo de Loja, Loja, Ecuador

## Abstract

Human papillomavirus (HPV) is the primary infectious agent for the development of cervical cancer, although the presence of the virus alone is insufficient for viral development and proliferation; this can be attributed to the increase in potential oncogenic risk, along with other risk factors. In the present investigation, the prevalence of high-risk HPV was determined from samples of premalignant or malignant cervical cytology in women from the southern region of Ecuador. The kit we used was able to detect genotypes 16, 18, 31, 33, 35, 39, 45, 51, 52, 56, 58, and 59. In addition, 64.5% of the analyzed samples were positive for HPV, with genotypes 16 and 18 being the most prevalent (16 was detected in 148 samples and 18 in 108). Genotypes 58 and 51 were the third most frequent simple and multiple infections, respectively. The data are very similar to those obtained worldwide, suggesting that the strategy of sex education, and the use of vaccines as primary prevention agents, could significantly decrease the incidence and mortality rate of cervical cancer in the southern region of Ecuador.

## 1. Introduction

According to data estimated for 2012, cervical cancer was the fifth most common type worldwide, behind breast, prostate, lung, and colorectal cancer. In the female population, it ranks fourth in incidence and mortality, with about 528,000 new cases and 266,000 deaths per year [1].

Data from the National Institute of Statistics and Censuses of Ecuador (INEC) that year found that cervical cancer was the second cause of death in women, due to oncological disease, with about 697 deaths, after stomach cancer [2].

In the southern region of the country, in the province of Loja, cervical cancer was reported as the most common cancer in women, with a total of 844 new cases diagnosed between 1997 and 2006. Loja province corresponds to 422 new cases, representing 23.1% of all malignant tumors in women. Of these 422 cases, 182 (43.1%) were carcinomas in situ. The standardized rate of the global incidence of invasive cervical cancer in this region was 31.5 × 100,000 inhabitants; in the case of in situ cancer, it was 23.8 × 100,000 inhabitants. Cervical cancer is also the number one cause of female death in the city of Loja, due to oncological disease, with a standardized rate of 9.6 × 100,000 inhabitants, identifying this disease as a major health problem in the southern region of Ecuador [3].

HPV is considered the main risk factor for the development of cervical cancer [4–7]. It is estimated that more than 50–75% of sexually active women are infected with one or more HPV genotypes throughout their lives [8, 9]. Once the infection occurs, the immune system achieves spontaneous regression [10, 11] in 80–90% of cases after 18–24 months [8, 12–14]. In this way, it is demonstrated that HPV infection is a necessary but insufficient reason for development of cancer [15, 16].

More than 150 HPV genotypes have been described. Approximately 40 of them have a special tropism via the anogenital and mucosal region [17–20]. They are classified into high oncogenic risk groups (16, 18, 31, 33, 34, 35, 39, 45, 51, 52, 56, 58, 59, and 68), possible oncogenic risk (26, 30, 34, 53, 66, 67, 69, 70, 73, 82, 85, and 97), and low-risk group that cause benign lesions like cervical warts or condylomas (6, 11, 28, 32, 44, 43, 44, 54, 55, 57, 61, 62, 71, 72, 74, 81, 83, 84, 86, 87, and 89) [6, 20–24]. Among high-risk genotypes, the most common worldwide are 16 and 18, accounting for approximately 70% of cases of cervical cancer, including high-grade squamous intraepithelial lesions [16, 25–28]. Several factors (related to a high probability of developing HPV infection) are age at first intercourse and having several sexual partners [28–31].

The main objective of the present study is to determine the prevalence of high-risk HPV in samples of premalignant or malignant cervical cytology in women from the southern region of Ecuador. The kit was able to detect the presence of genotypes 16, 18, 31, 33, 35, 39, 45, 51, 52, 56, 58, and 59. Papanicolaou test samples were collected according to the Bethesda Classification Diagnosis of any cellular epithelial abnormality. In addition, the possible association of different risk factors with HPV infection was evaluated. Given the high incidence and mortality rate of cervical cancer, and the absence of respective epidemiological studies in this area, the study may contribute to a reduction in its number of deaths.

## 2. Population and Methods

### 2.1. Population

This observational, cross-sectional, and prospective study was carried out from 2012 to 2013, having been approved by the research committees of the Universidad Técnica Particular de Loja (UTPL) and the Cancer Society of Ecuador (SOLCA) Store core. This study was supported by Universidad Técnica Particular de Loja: Projects PY1019 and PY1620.

A total of 431 women between 17 and 84 years of age used the gynecological services of the SOLCA Hospital, the UTPL Hospital, or the Ecuadorian Family Well-Being Association (APROFE) in the city of Loja, Ecuador, with the intention of learning their Pap test results.

*Inclusion Criteria*

- Women domiciled in the Province of Loja, Zamora, or El Oro
- Cytological diagnosis of the premalignant or malignant pap smear (ASC-US, ASC-H, LSIL, HSIL, squamous cell carcinoma, AGC, or adenocarcinomas), which had been previously untreated
- Performing the diagnostic colposcopy
- Acceptance of participation in the study through signing an informed consent

*Exclusion Criteria*

- Women who have given birth or had a Pap smear in the last 3 weeks
- Women who underwent colposcopy in the last 6 weeks
- Women who have undergone an invasive procedure in the uterine cervix (conization, biopsy, etc.) in the last 3 months

### 2.2. Methods

The sociodemographic data (age, place of residence, and marital status) and clinical data (age at first intercourse, number of partners, and history of sexually transmitted infections) of the volunteers were collected by survey.

Patients in the study underwent a cervical scraping, with a sterile cytobrush at the start of the diagnostic-therapeutic colposcopy. Samples were stored in 5 ml of PBS buffer (pH 7.5) between 6 and 8°C for a maximum period of 3 days until processing.

DNA extraction was performed with a commercial kit (Pure Link DNA, Invitrogen Co., Grand Isle, NY, USA). The quantity and quality of the DNA were measured by spectrophotometry, using the Nanodrop 200c, Thermo Scientific equipment. It was carried out by examining its optical density of 260/280-260/230 nm. Between 10 and 100 ng/*μ*L of total DNA extracted for amplification was used.

HPV detection was done by real-time PCR, using the AmpliSens HPV genotype FTR kit (Bretonneux, FR) following the manufacturer's instructions. The kit recognizes 12 high-risk HPV genotypes (16, 18, 31, 33, 35, 56, 39, 45, 51, 52, 58, and 59). Positive and negative controls were used for each reaction. Amplification of the *β*-globin gene was a quality control measure of the genetic material.

The data obtained via amplification were analyzed by the Applied Biosystems Fast 7500 Software Detection System (Thermo Fisher Scientific, Waltham, MA, USA). We used a system of presence-absence detection of the genetic material for the virus with 4 fluorophores Cy5 (for *β*-globin), FAM (for genotypes 16, 39, 33, and 58), JOE (for genotypes 31, 45, 35, and 52), and ROX (for genotypes 18, 59, 56, and 51). The cycle threshold (CT) was 35, according to the manufacturer's instructions. Samples that exceeded this value were considered positive, by independently observing the behavior of the amplification curve of each genotype.

### 2.3. Statistical Analysis

The results were analyzed with descriptive statistics, identifying mean values, standard deviation, and/or percentages as appropriate. To assess the association of different risk factors and the presence of HPV infection, the OR (95% CI) was calculated, considering significant values as *p* < 0.05. Calculations were made with these programs: IBM SPSS Statistics (IBM, Armonk, NY, USA) and MedCalc® statistical software (Ostend, BE).

## 3. Results

Characteristics of the 431 participants in the study are shown in Table 1. Furthermore, 64.5% (278) of the samples tested positive for HPV. Genotypes 16 and 18 were the most prevalent, with 16 being detected in 148 samples (53.2% of positive samples) and 18 detected in 108 (38.8%). Another 110 samples (39.6%) involved a single viral type, while 168 samples (60.4%) had multiple infections in samples with up to 7 different genotypes.  Table 2 shows the frequency of each type of HPV infection, considering single and multiple displays.

Considering the cytological diagnosis and presence of HPV, 66.7%, 61.7%, 52.4%, 63.3%, 67.5%, and 86.7% of the samples with AGUS, ASCUS, ASC-H, LIE-AG, and cancer, respectively, had high-risk HPV. The first three HPV genotypes most frequent in each cytologic diagnosis are shown in Table 3.

The association analysis between HPV infection and the risk factors studied, as well as the OR values, is shown in Table 4.

## 4. Discussion

In this first study of its kind for HPV genotypes, we determined the frequency of premalignant and malignant samples of cervical cytology, along with their association with cytological diagnosis and risk factors of infection. The most frequent cytopathology was LSIL at 36.7%, while cancer cases accounted for 3.5% of total samples. The prevalence of high-risk HPV in cytological samples was 64.5%. In Quito, Ecuador, they reported other studies in cytological and/or histologically altered, in which the reported prevalence of HPV was found to be low risk at 67.7% and 86% [32, 33]. In the city of Cuenca, in a study of 500 women from the general population, the prevalence of 25.6% of HPV for both high and low risk [34] was similar to that reported in Santa Elena. Brown et al. (2009) found [35] a prevalence of 24.2%. A higher prevalence of HPV with abnormal cytology studies indicates a relationship between the virus and malignant cells.

In the present study, genotypes 16 and 18 were the most frequent, for both simple infections (47.3% and 15.5%, resp.) and multiple infections (57.1% and 54.2%, resp.). Other oncogenic types showed significant incidence. Genotype 58 was presented as the third most common in simple infections (10%), while 51 ranked third in multiple infections (42.9%). These results agree with the meta-analysis of Bruni et al. (2010) [36], which reports genotypes 16, 18, 52, 31, and 58 as the most frequent. In the systematic review and meta-analysis of Ciapponi et al. (2011) [37], the prevalence of HPV in high-grade lesions and cancer of the uterine cervix in Latin America and the Caribbean (analyzing nearly 8,000 samples) was most frequently found in genotypes 16 and 18, followed in descending order by HPV 31, 58, 33, 45, and 52, similar to those obtained in our own study data. Mexico reported the 58 genotype as the second most common after HPV 16 in cervical cancer and abnormal cytology samples [38, 39] and, in Quito, in altered cervical biopsy samples [32].

Studies in Ecuador found genotype 16 as the most common [32, 33, 35, 40], along with results of this investigation. The 18 genotype was not reported as the second most frequent, suggesting that genotypic variability by geographic region and sociodemographic factors had an effect, such that more research is necessary.

Considering the cytological diagnosis, the three most frequent HPV genotypes were 16, 18, and 51 in AGUS, ASC-H, LSIL, HSIL, while in cancer and ASCUS the type 58 was the third most frequent, behind genotypes 16 and 18. Some authors suggest that the presence of these genotypes (high-grade lesions) would be more likely to progress to cervical cancer than other HPV types; this is due to the oncogenic potential of these genotypes [41].

There are several risk factors that can influence HPV infection. Some researchers have reported that early onset of sexual life and a higher number of sexual partners significantly increased risk of infection [42, 43]. Some studies indicated that single or divorced women were more likely to have cervical cancer; however, according to reports in Latin America, men tend to have more than one sexual partner, limiting the use of marital status as a risk factor for contracting this virus [44]. To reduce risk of infection, condom use is recommended for sex, and, in some studies, it could reduce the risk of HPV infection by up to 70% [45, 46]. However, there was no significant association in our study for increased risk of infection, given the factors analyzed; this could be due to a possible bias in the information provided by the participants, due to sociocultural factors; in addition, these questions are based on memory and a large number of people did not answer the questions, affecting significantly the results obtained.

In conclusion, in this study, genotypes 16 and 18 were identified as the main types of HPV in the samples analyzed for premalignant and malignant cytology. Genotypes 58 and 51 were presented as the third most frequent type, considering simple and multiple infections, respectively. The data are very similar to those obtained worldwide, again suggesting that the common strategies of sex education and vaccine use as primary preventions could significantly decrease the incidence and mortality rate of cervical cancer in the southern region of Ecuador. Particularly it is considered as potentially beneficial to introduce the integral sexual education in the primary school for 10-11-year-old children because of early beginning of sexual intercourse in the Ecuadorian population.

## Figures and Tables

**Table 1:** Characteristics of participants.

Characteristics	Values
*Age*	
Range	17–84
Average (SD)	40.7 (13.5)
*Age of onset of sexual life *	
Range	10–45
Average (SD)	18.9 (4.3)
Does not answer (%)	29 (6.7)
*Number of sexual partners*	
Range	1–10
Average (SD)	1.6 (1.0)
Does not answer (%)	2 (0.5)
*Age at first pregnancy*	
Range	11–39
Average (SD)	20.8 (4.6)
Does not answer (%)	218 (50.6)
*Number of pregnancies*	
0 (%)	46 (10.7)
<3 (%)	159 (36.9)
≥3 (%)	224 (52.0)
Does not answer (%)	2 (0.5)
*Condom use*	
No (%)	335 (77.7)
Yes (%)	49 (11.4)
Does not answer (%)	47 (10.9)
*Civil status*	
Married (%)	285 (66.1)
Divorced (%)	21 (4.9)
Single (%)	71 (16.5)
Does not answer (%)	54 (12.5)
*Level of education *	
Primary (%)	68 (15.8)
High school (%)	73 (16.9)
College (%)	87 (20.2)
Does not answer (%)	203 (47.1)
*Learn about HPV*	
No (%)	331 (76.8)
Yes (%)	71 (16.5)
Does not answer (%)	29 (6.7)
*Abnormal cytology*	
AGUS (%)	60 (13.9)
ASCUS (%)	94 (21.8)
ASC-H (%)	21 (4.9)
LSIL (%)	158 (36.7)
HSIL (%)	83 (19.3)
Cancer (%)	15 (3.5)

**Table 2:** Frequency of HPV genotypes in positive cases.

Genotype	Simple infection		Multiple infection	
	*n*	%^a^	*N*	%^a^
HPV 16	52	47.3	96	57.1
HPV 18	17	15.5	91	54.2
HPV 31	3	2.7	41	24.4
HPV 33	1	0.9	11	6.6
HPV 35	0	0.0	11	6.6
HPV 39	2	1.8	23	13.7
HPV 45	0	0.0	7	4.2
HPV 51	9	8.2	72	42.9
HPV 52	1	0.9	27	16.1
HPV 56	5	4.6	27	16.1
HPV 58	11	10.0	63	37.5
HPV 59	9	8.2	42	25.0

^a^The percentage is calculated relative to the total positive samples of simple infections (110) and multiple infections (168), respectively.

**Table 3:** Frequency of HPV genotypes in all cytological diagnoses.

Cytology (HPV cases)	Genotype	*n* ^a^	%^b^
AGUS (40)	HPV 16	23	57.5
	HPV 18	20	50.0
	HPV 51	11	27.5
	Other high-risk HPV^c^	39	97.5

ASCUS (58) 61.70%	HPV 16	29	50.0
	HPV 18	23	39.7
	HPV 58	15	25.9
	Other high-risk HPV^d^	56	96.6

ASC-H (11) 52.38%	HPV 16	4	36.4
	HPV 18	4	36.4
	HPV 51	4	36.4
	Other high-risk HPV^c^	11	100.0

LSIL (100) 63.29%	HPV 16	49	49.0
	HPV 18	35	35.0
	HPV 51	34	34.0
	Other high-risk HPV^c^	122	122.0

HSIL (56) 67.47%	HPV 16	35	62.5
	HPV 18	19	33.9
	HPV 51	15	26.8
	Other high-risk HPV^c^	43	76.8

Cancer (13) 86.67%	HPV 16	8	61.5
	HPV 18	7	53.9
	HPV 51	3	23.1
	HPV 56	3	23.1
	Other high-risk HPV^e^	10	76.9

^a^Simple and multiple infections together. ^b^Percentage in total positive samples of each cytological alteration. ^c^HPV 31, 33, 35, 56, 39, 59, 52, 58. ^d^HPV 31, 33, 35, 56, 39, 45, 59, 51, 52. ^e^HPV 33, 35, 39, 45, 59, 52, 58.

**Table 4:** Risk factors and HPV infection.

Variables	Positives	Total	OR (IC 95%)
*Age of onset of sexual life*			
≥21	65	97	
<21	195	305	0.87 (0.5–1.4)
*Number of pregnancies*			
<3	136	205	
≥3	141	224	0.86 (0.6–1.3)
*Number of sexual partners*			
1	171	262	
>1	105	167	0.90 (0.6–1.4)
*Condom use*			
Yes	35	49	
No	218	335	0.75 (0.4–1.4)
*Civil status*			
Married	185	285	
Single-divorced	56	92	0.84 (0.5–1.4)
*Level of education*			
High School-college^a^	110	160	
Primary	55	68	1.92 (0.9–3.8)
*Learn about HPV*			
Yes	51	71	
No	211	331	0.69 (0.4–1.2)

^a^Primary education level included 7 years of study, and secondary level of education included over 7 years of study.
